# Time course study of the response to LPS targeting the pig immune gene networks

**DOI:** 10.1186/s12864-017-4363-5

**Published:** 2017-12-22

**Authors:** Elena Terenina, Valérie Sautron, Caroline Ydier, Darya Bazovkina, Amélie Sevin-Pujol, Laure Gress, Yannick Lippi, Claire Naylies, Yvon Billon, Laurence Liaubet, Pierre Mormede, Nathalie Villa-Vialaneix

**Affiliations:** 1 INRA, UMR 1388 GenPhySE, Université de Toulouse, INRA, INPT, ENVT, Castanet-Tolosan, F-31326 France; 2 Department of Behavioral Neurogenomics, Siberian Branch of the Russian Academy of Sciences, Novosibirsk, 630090 Russia; 3 Toxalim (Research Centre in Food Toxicology), Université de Toulouse, INRA, ENVT, INP-Purpan, UPS, Toulouse, F-31027 France; 4 INRA, UE 1372 GenESI, Surgeres, F-17700 France; 5 MIAT, Université de Toulouse, INRA, Castanet-Tolosan, France

**Keywords:** Stress, Hypothalamic-pituitary-adrenal (HPA) axis, Cortisol, Time-course, Systems biology, Microarray, Pig

## Abstract

**Background:**

Stress is a generic term used to describe non-specific responses of the body to all kinds of challenges. A very large variability in the response can be observed across individuals, depending on numerous conditioning factors like genetics, early influences and life history. As a result, there is a wide range of individual vulnerability and resilience to stress, also called robustness. The importance of robustness-related traits in breeding strategies is increasing progressively towards the production of animals with a high level of production under a wide range of climatic conditions and management systems, together with a lower environmental impact and a high level of animal welfare. The present study aims at describing blood transcriptomic, hormonal, and metabolic responses of pigs to a systemic challenge using lipopolysaccharide (LPS). The objective is to analyze the individual variation of the biological responses in relation to the activity of the HPA axis measured by the levels of plasma cortisol after LPS and ACTH in 120 juvenile Large White (LW) pigs. The kinetics of the response was measured with biological variables and whole blood gene expression at 4 time points. A multilevel statistical analysis was used to take into account the longitudinal aspect of the data.

**Results:**

Cortisol level reaches its peak 4 h after LPS injection. The characteristic changes of white blood cell count to LPS were observed, with a decrease of total count, maximal at *t*=+4 h, and the mirror changes in the respective proportions of lymphocytes and granulocytes. The lymphocytes / granulocytes ratio was maximal at *t*=+1 h. An integrative statistical approach was used and provided a set of candidate genes for kinetic studies and ongoing complementary studies focused on the LPS-stimulated inflammatory response.

**Conclusions:**

The present study demonstrates the specific biomarkers indicative of an inflammation in swine. Furthermore, these stress responses persist for prolonged periods of time and at significant expression levels, making them good candidate markers for evaluating the efficacy of anti-inflammatory drugs.

**Electronic supplementary material:**

The online version of this article (doi:10.1186/s12864-017-4363-5) contains supplementary material, which is available to authorized users.

## Background

Over time, farms have evolved towards factory production units. This has led to a decline of the welfare of animals that becomes an important concern for consumers [1]. Moreover, this type of farming has led to the selection of animals with high production traits such as rapid growth, lean meat, or large litters. However, the strong selection focus on these characteristics is suspected to reduce functional traits, such as viability of the newborns or disease resistance. Consequently, the genetic potential of animals is usually not fully expressed in commercial conditions, due to the limiting influence of the environment. Robustness is a specific quality of an individual to express a high production potential in a wide variety of environmental conditions and is now a major specific breeding goal in the context of sustainable farm animal breeding. Various strategies are available to increase robustness, and we have suggested that the reinforcement of the neuroendocrine stress responses may favour the processes of adaptation and dampen the negative consequences of the environment [2]. The hypothalamic-pituitary-adrenocortical (HPA) axis is the main neuroendocrine system involved in adaptation to stress and is strongly influenced by genetic factors [3]. It is therefore a primary candidate for the selection of more robust animals [2].

In modern intensive livestock production, pigs are easily threatened by different types of inflammation. Immunological stress is a comprehensive process involving immunological, neurological, and endocrinological responses [4]. The reciprocal “subjugation” of the brain and the immune system via cytokines and stress hormones is now well demonstrated [5, 6]. The resulting balance has more recently been demonstrated at the level of blood cell transcriptome [7], with chronic stress increasing the expression of genes regulated by inflammatory mediators and decreasing those regulated by glucocorticoid hormones [8]. This approach has been used to evaluate the negative consequences of adverse environmental conditions, mostly in humans but also in farm animals (horses [9]). More recently, individual differences have also been described as related to personality dimensions in humans [10]. However the relationships with individual variations of HPA axis activity, including genetic factors, is still unexplored.

We have previously shown large variations in biological and transcriptomic responses to an ACTH stimulation test [11]. Indeed, the adrenal response to ACTH is a major source of variability of HPA axis function [12]. The present study aims at describing blood transcriptomic, hormonal, and metabolic responses of pigs to a systemic challenge using lipopolysaccharide (LPS), a major component of the outer membrane in gram-negative bacteria [13]. LPS provokes an acute inflammatory syndrome resulting eventually in all kinds of pathophysiological damages [14]. The objective is to analyze the individual variation of the biological responses in relation to the activity of the HPA axis. This was assessed through the level of cortisol released by LPS (this experiment) and also, in the same animals, through the level of cortisol released after an ACTH stimulation test (in an experiment previously published [11]).

## Methods

### Animals, treatment and blood sampling and biological analyses

The same 120 piglets (63 females and 57 males) as described in [11] were used for this study. In addition to the ACTH stimulation test, previously described, each animal was injected in the neck muscles with LPS at 8 weeks (*E. coli* serotype 055:B5, Sigma-Aldrich, Saint Quentin Fallavier, FR) at a dose of 15 *μ*g/kg body weight. Injections occurred from 10:00-11:00 AM to avoid nycthemeral variations. Blood samples were collected before the injection (*t*=0) and 1 h (*t*=+1), 4 h (*t*=+4) and 24 h (*t*=+24) after injection with the same protocol as described in [11].

Cortisol, glucose, free fatty acid (FFA), blood cell counts (including: white cells count, proportion of lymphocytes, monocytes and granulocytes, red cells count, hematocrit, concentration of hemoglobin, red cells width and volume, platelets count and platelets width and volume) were obtained using the same protocol as in the previous study. Fifteen biological variables were measured on the 120 pigs in addition to birth and weaning weights. All these variables were preprocessed for outlier and missing value correction and to ensure normality as in the previous study.

### Whole blood transcriptome

A subset of 30 females from 2 batches only was used to study pangenomic expression in whole blood cells at each time point (120 samples). Total RNA isolation and purification was done as described in [11].

Gene expression analysis was performed at the GeT-TRiX facility (GénoToul, Génopole Toulouse Midi-Pyrénées) using Agilent SurePrint G3 porcine microarray GPL16524 (Agilent, 8 ×60 K) following the manufacturer’s instructions (Agilent Technologies, Santa Clara, California). For each of 120 samples, Cyanine-3 (Cy3) labeled cRNA was prepared from 200 ng of total RNA using the One-Color Quick Amp Labeling kit (Agilent) according to the manufacturer’s instructions, followed by Agencourt RNAClean XP (Agencourt Bioscience Corporation, Beverly, Massachusetts). 600 ng of Cy3-labelled cRNA were hybridized on the microarray slides following the manufacturer’s instructions. Immediately after washing, the slides were scanned on Agilent G2505C Microarray Scanner using Agilent Scan Control A.8.5.1 software and fluorescence signal extracted using Agilent Feature Extraction software v10.10.1.1 with default parameters (grid 037880_D_F_20120213 and protocol GE1_1010_Sep10).

### Hybridization protocol

Blood samples of 2 pigs at one time step each were of poor quality and thus not used. The same experimental design than the one described in [11] was used to secure the kinetics of the response for each individual and to prevent confounding effects between batch and array. After quality control and filtering, 27,837 probes were kept and log2 transformed. Technical biases and missing data were handled similarly than in the previous study.

### Fluidigm Biomark RT-PCR

For validation of array data by Fluidigm technology 22 animals (88 samples) were kept to fit the technical constraints of this technique. Total RNA (1 *μ*g) used in microarray experiments was reverse-transcribed as previously described [15]. Primer sequences for genes were designed using Primer3plus software (http://primer3plus.com) and are given in Additional file 1. The *TFRC* gene (transferrin receptor) and *EPRS* gene (glutamyl-prolyl-tRNA synthetase) were used as internal controls. Pre-amplified samples were analyzed with a 96 ×96 Dynamic Array™ IFC (Fluidigm) following the protocol defined by [16], with some modifications. All measurements were performed on the same plate. Each gene was tested twice for each sample. Four dilution points containing a pool of all samples were used to determine PCR efficiency. Data were analyzed using BioMark Gene Expression Data Analysis software (Fluidigm) to obtain Ct values. The Pfaffl method was applied to compute the relative expression of each gene [17]. Pearson correlations were computed to compare the expression values of microarray and quantitative real-time PCR. Quantitative RT-PCR data were also analyzed for time effect by ANOVA with repeated measurements for every gene.

## Statistical analyses

All analyses were performed with the R software, version 3.2.2 [18]. They were designed so as to address two main questions: the first one is the study of the evolution over time of the different variables (plasma metabolites, cortisol and gene expression) after LPS injection. The second one is the study of the relation between the different variables and one of the most relevant measure of sensitivity of the adrenals, the cortisol level one hour after ACTH injection (data from [11], obtained on the same animals).

Longitudinal data analysis of the evolution over time of the different variables can be performed using different statistical methods. A very common approach is to fit curves (for instance splines as in [19, 20]) a as prior processing to the statistical analysis. However, four time steps are too few number of time points to obtain an accurate fit. The analysis was thus performed using two main approaches: the first one relies on linear models with the time as a factor covariate and the second one is based on a decomposition of sources of variations, as was already proven successful for repeated measurements analysis in [21] and for longitudinal data analysis in [11].

### Statistical analysis of plasma metabolites and cortisol

**Fig. 1:**
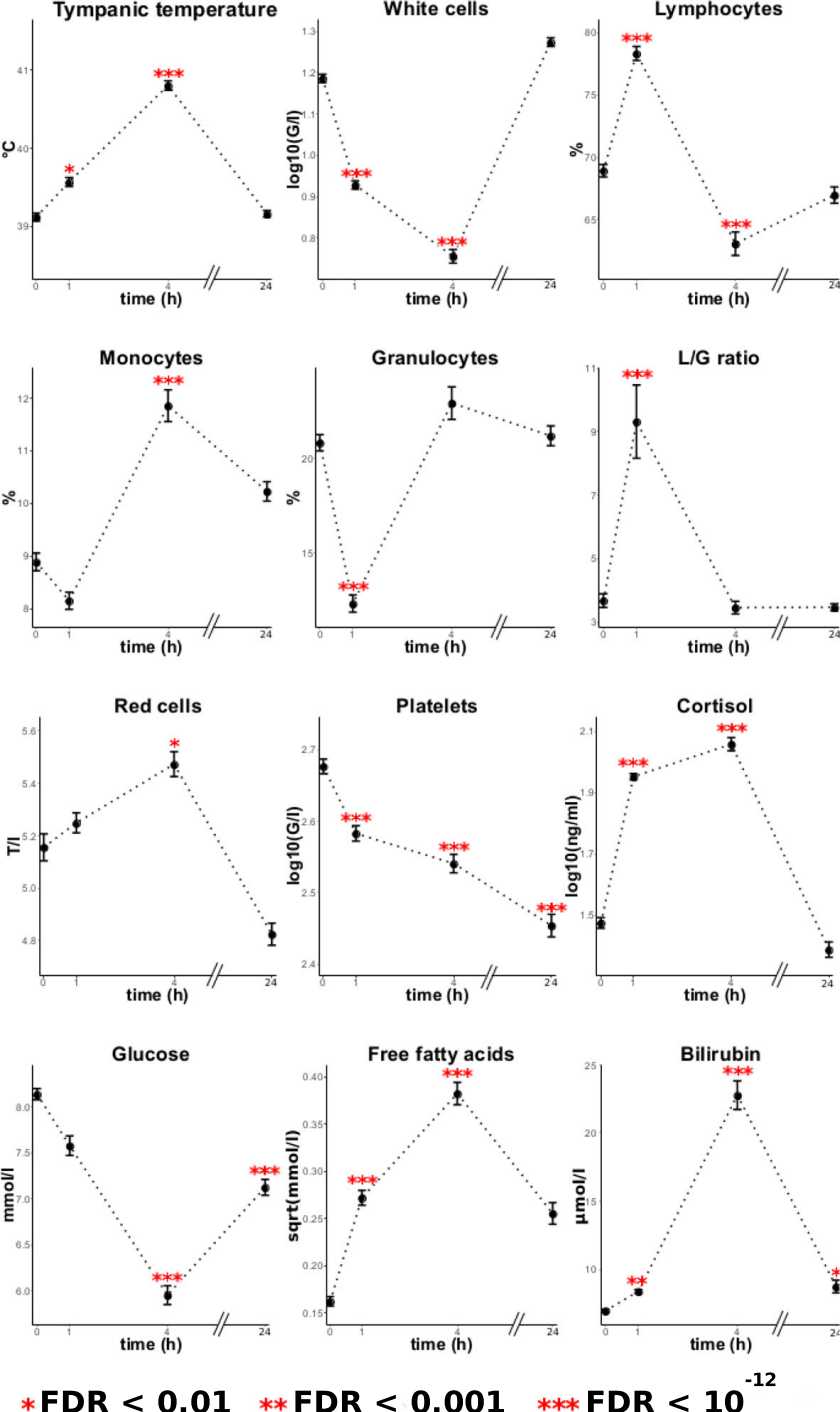
Mean evolution of the biological variables over time. Vertical bars correspond to + and - SEM at each point

First, all variables were subjected to a one-way ANOVA with repeated measures and the time step as a factor covariate. In order to control the false discovery rate (FDR) [22], *p*-values were adjusted using a Benjamini-Hochberg (BH) approach (Table 1). Variables with an adjusted *p*-value (FDR <0.05) were then subjected to 3 paired t-tests to assess the difference between *t*=0 and *t*=+1, between *t*=0 and *t*=+4 and between *t*=0 and *t*=+24. The full list of *p*-values was adjusted using a BH approach (Fig. 1).

**Table 1:** Reference values (at *t*=0) for the biological variables, birth weight and weaning weight (*n*=120)

	Units	Min	Max	Mean	SEM	*F*
Tympanic temperature	°C	36.100	40.257	39.168	0.050	258.110
White cells	log10(G/l)	0.491	1.472	1.188	0.011	572.970
Lymphocytes	%	46.600	91.900	67.477	0.555	112.180
Monocytes	%	3.900	16.200	8.557	0.191	69.500
Granulocytes	%	2.500	35.600	22.608	0.527	78.210
*L*/*G* ratio		1.355	36.760	3.466	0.295	64.650
Red cells	T/l	1.490	7.330	5.163	0.054	69.420
Mean corpuscular volume	fl	39.700	63.700	52.008	0.333	62.120
Hematocrit	%	6.800	37.400	26.828	0.299	78.990
Hemoglobin	g/dl	6.900	12.800	8.947	0.092	46.680
Red blood cells distribution width	fl	29.100	33.800	32.029	0.081	74.440
Platelets	log10(G/l)	2.330	2.998	2.667	0.011	227.400
Mean platelet volume	fl	7.600	13.000	9.682	0.102	71.210
Platelet distribution width	%	9.600	12.000	10.771	0.045	122.790
Cortisol	log10(ng/ml)	1.041	2.033	1.475	0.017	370.240
Free fatty acids	$\sqrt {(\text {mmol/l})}$	0.079	0.560	0.162	0.005	111.040
Glucose	mmol/l	5.850	9.525	8.035	0.061	123.990
Bilirubin	*μ*mol/l	4.660	13.000	8.523	0.190	178.610
Birth weight	kg	0.400	2.680	1.492	0.033	
Weaning weight	kg	5.460	16.564	9.486	0.174	

Results of the ANOVA for time effect (*F* value): all variables varied significatively during the experiment, with an FDR <10^−12^, except for the weights (not tested because constant)

In addition, the influence of sex on the biological variables was tested using a two-way ANOVA with repeated measures including sex as a covariate. *p*-values were adjusted using a BH approach.

Cortisol levels measured one hour after ACTH injection are the most relevant measure to assess the sensitivity of the adrenals to ACTH (data from [11]). Hence, correlations between biological variables at *t*∈{0,+1,+4,+24} and the level of cortisol one hour after ACTH injection were investigated using t-tests of the linear regression on ACTH level. *p*-values were adjusted using a BH approach.

### Statistical analysis of the transcriptome

#### Differentially expressed probes (DEP)

The whole blood is composed of different types of white cells with distinct roles which express different kinds of transcripts [23]. It is thus likely that a modification in blood cell composition may influence the gene expression level without having cells actually express transcripts differently. As blood cell composition was found to vary over time after LPS injection, we used the $\frac {\text {Lymphocyte}}{\text {Granulocyte}}$ (*L*/*G*) ratio as a covariate in our analyses.

Three different approaches were used to identify relevant probes. The first two are longitudinal analyses aiming, respectively, at identifying probes with an expression significantly varying from their basal levels after LPS injection and probes with a varying contribution of the L/G ratio to their expressions after LPS injection. The last analysis searched for probes correlated to the level of cortisol one hour after ACTH injection.

Firstly, we identified probes differentially expressed at each time step while taking blood cell composition into account. Blood cell composition was measured by the *L*/*G* ratio. Three models (one for each time step *t* ^′^ where *t* ^′^∈{+1,+4,+24}) were fitted to each probe using observations at *t*=0 and *t*=*t* ^′^.

1 $$  \text{expr}_{{it}} = \mu_{0} + \tau_{t'}\mathbb{I}_{\{t = t'\}} + \beta^{t'} L/G_{{it}} + \epsilon_{{it}}  $$

with *i*=1,…*n* is animal *i*. expr_*it*_ is the expression of the probe being studied for animal *i* at time step *t* (*t*∈{0,*t* ^′^}), *μ*_0_ is the specific contribution of time step *t*=0, $\phantom {\dot {i}\!}\tau _{t^{\prime }}$ is the effect of time step *t* ^′^, $\phantom {\dot {i}\!}\beta ^{t^{\prime }}$ is the effect of *L*/*G* ratio in this model and $\epsilon _{it} \sim N\left (0, \sigma _{e}^{2}\right)$ is an error term.

We then tested the contribution of time step *t* ^′^ against the null hypothesis *H*_0_: $\phantom {\dot {i}\!}\tau _{t^{\prime }} = 0$. The full list of *p*-values was globally adjusted using a Bonferroni approach. As the Bonferroni approach exerts a more stringent control than the BH approach, it was used to obtain a narrowed list of the most significant probes. Probes with at least one adjusted *p*-value <0.01 were probes for which the expression adjusted by the *L*/*G* ratio was significantly different from the basal level. In the sequel, this list of genes will be referred to as (List1).

Secondly, we identified probes for which the *L*/*G* ratio effect is different according to the time step. To that aim, we compared a complete model, including all time step contributions and the *L*/*G* ratio effect according to the time step (Eq. (2)):

2 $$  \text{expr}_{{it}} = \tau_{t} + \beta_{t} L/G_{{it}} + \epsilon_{{it}}  $$

(with *t*∈{0,1,4,24} and *β*_*t*_ is the interaction effect between time step *t* and the *L*/*G* ratio of individual *i* at time step *t*), to a reduced model, including only the average *L*/*G* ratio and all time step contributions (Eq. (3)):

3 $$  \text{expr}_{{it}} = \tau_{t} + \beta L/G_{{it}} + \epsilon_{{it}}.  $$

An F-test was then performed to test the null hypothesis, *H*_0_: *β*_0_=*β*_1_=*β*_4_=*β*_24_, against the alternate hypothesis, *H*_1_: ∃*t*_1_,*t*_2_ such as $\beta _{t_{1}} \neq \beta _{t_{2}}$. Multiple testing was handled by applying a BH approach (FDR <0.05). Probes for which the test was significant were probes for which the effect of *L*/*G* varied over time. In the sequel, this list of genes will be referred to as (List2).

Finally, we studied correlations between all probes and cortisol level when it reaches its peak in blood after LPS injection. Thus, Pearson correlations, *ρ*, were computed between DEP expression at each time step and cortisol level at *t*=+4. A correlation test was then performed to test the null hypothesis, *H*_0_: *ρ*=0 against *H*_1_: *ρ*≠0. Multiple testing was handled by using a BH approach (FDR <5*%*). This list of genes will be referred as (List3) in the sequel.

In addition, to link probes responding to a LPS injection with a measure of the HPA axis activity, we studied correlations between expression of all probes and the cortisol level at one hour after ACTH injection, as measured on the same pigs in [11].

All lists of DE probes were then annotated and duplicated probes were removed by keeping only DEP with the smallest FDR per annotated gene and all non-annotated genes. Remaining genes will be referred as differentially expressed genes (DEG) in the sequel.

### Functional analysis

Enrichment analysis was performed using tools available at WEB-based GEne SeT AnaLysis Toolkit (WebGestalt) [24, 25]. Entrez gene IDs were used as unique gene identifiers. Target gene lists for main effects and interactions and a background gene set consisting of all 9530 genes were used to identify enrichment in GO, KEGG, Transcription Factor Target, Microarray Target, Protein Interaction Network Module, and Phenotype Analysis in WebGestalt using Fisher’s exact test and BH correction for multiple testing.

A pathway is an interconnected arrangement of processes, representing the functional roles of genes in the genome. The biological processes in which individual genes may participate were identified using the “Gene Ontology” database AmiGO (http://amigo.geneontology.org). The DEG were assembled into networks using Ingenuity Pathway Analysis (IPA ^Ⓒ^) (http://www.ingenuity.com). This application includes algorithms that automatically identify the biological pathways and functions of selected genes. It is based on a large bibliographic database with various types of interaction already identified between pairs of genes. Every biological network extracted by IPA corresponds to the best possible arrangement of the genes, and are associated with a score derived from a *p*-value (right-tailed Fisher’s exact test, − log10-transformed).

### Time course analyses

In the case of time course analyses, the approach previously described (applying a univariate linear model on each variable followed by multiple test correction) is common. However, this approach disregards the dependencies between genes and does not allow for a global view of the relationships between the repeated measurements in high dimensional data. The multilevel approach, already proven successful to investigate the relationships between repeated measurements in [21] was thus used and combined with multivariate data analysis methods.

The multilevel approach [21] is inspired by the mixed-model framework and uses a split-up variation of the (*n* *T*)×*p* matrix *X* that contains the observations of *p* variables (clinical biology variables or gene expressions) on *n* animals with *T*=4 times of measurements:

4 $$ {\begin{aligned} X = \underbrace{X_{..}}_{\textrm{offset term}} + \underbrace{X_{b}}_{\textrm{between-animal deviation}} + \underbrace{X_{w}}_{\textrm{within-animal deviation}} \end{aligned}}  $$

Similarly to what was performed in [11], multivariate approaches were performed on *X*_*w*_ to bring out the most relevant correlations between variables in the dataset, independently from individual variations. First a multilevel PCA was performed on the biological variables to study the overall effect of LPS on plasma metabolites and cortisol over time. Then, a multilevel multiple factor analysis (MFA) [26] was used to investigate the overall relationships between clinical biology and transcriptomic data.

## Results

### Plasma cortisol, metabolites, and blood cell counts

Baseline values of biological variables and the global time effect, and birth and weaning weights are shown in Table 1. Figure 1 shows the evolution of the main variables over time.

Tympanic temperature peaked at *t*=+4 (40.8 °C vs 39.1 °C) and returned to basal levels at *t*=+24. A decrease of total count of white blood cell count was observed, maximal at *t*=+4 (5.70 vs 15.35 G/l) and the mirror changes in the respective proportions of lymphocytes and granulocytes. This indicated that the lymphocytes/granulocytes ratio (*L*/*G*) was a good measure to use in order to take into account these changes that result mainly from the redistribution of lymphocytes into the tissues [27]. The *L*/*G* ratio was maximal at *t*=+1 (9.32 vs 3.67) and back to basal levels at *t*=+4. The red blood cell count and associated measures (hematocrit and hemoglobin concentration) showed a biphasic change, with an initial increase, maximal at *t*=+4 (5.47 vs 5.16 T/l) and a subsequent long-lasting decrease (4.82 T/l at *t*=+24). The platelet count showed a steady decrease until at least *t*=+24 (284 vs 475 G/l). These measures were not influenced by sex, except the mean red cell volume and hematocrit that were slightly lower in males (FDR <0.05).

Cortisol levels peaked at *t*=+4 with a 3.83-fold increase (114.3 vs 29.8 ng/ml). Circulating glucose levels were reduced by 26.9% to 5.95 mmol/l at *t*=+4. The circulating concentration of free fatty acids increased from 0.026 to 0.146 mmol/l at *t*=+4. None of these biochemical measures was influenced by sex.

### Overall effect of LPS on clinical biological variables

**Fig. 2:**
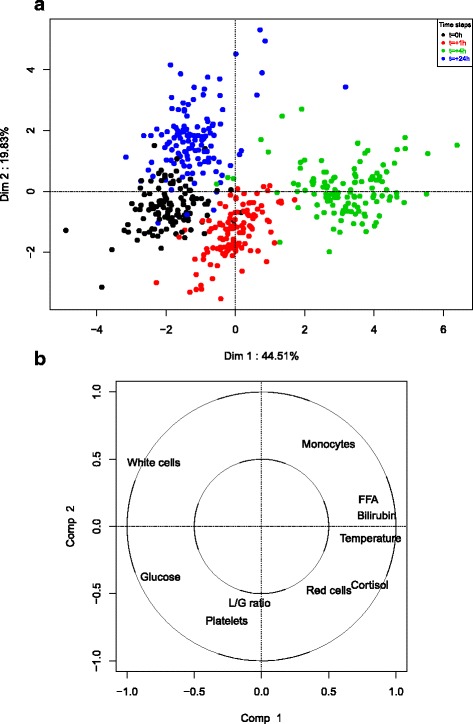
Multilevel PCA on the biological variables responding to LPS. Colors represent the time step; Black: *t*=0; Red: *t*=+1; Green: *t*=+4; Blue: *t*=+24; **a**: Projection of the individuals on dimensions 1 - 2; **b**: Projection of the variables on dimensions 1 - 2

The overall effect of LPS over time was investigated with a multilevel PCA (Fig. 2). The first component of the multilevel PCA opposes the observations at *t*=0 (negative coordinates on this axis) to the observations at *t*=+4 (positive coordinates on this axis), this time step corresponding to the peak of LPS effect. The second component opposes the observations at *t*=+24 (positive coordinates on this axis) to the other observation times (negative coordinates on this axis). The representation of the variables shows that the first axis is mainly driven by an opposition between free fatty acids (FFA), bilirubin, temperature and cortisol (high measures at *t*=+4), and white cell count and glucose (low measures at *t*=+4). The second axis of the PCA is driven by *L*/*G* ratio and platelet count that are high at *t*=+1.

No biological variable was found to be correlated to cortisol level one hour after ACTH injection.

### Differentially expressed genes related to key immune functions

In our study, we used a comprehensive gene expression profiling by means of microarray analysis to identify clusters of genes differentially expressed in peripheral blood cells, taking into consideration the kinetic of the response with 4 time points (*t*∈{0,+1,+4,+24}). LPS induces dramatic changes in blood cell number and lymphocyte/granulocyte (*L*/*G*) ratio that introduces a confusion between time and cell type effects, and a major challenge for the interpretation of transcriptomic data. Therefore we based the interpretation of the results on three different lists of genes, (List1), (List2), and (List3).

All genes found to be differentially expressed after ACTH injection in our previous study [11] were also found in one of these three lists.

### Analysis of each list of genes

The **first list of genes (List1)** consists of 9530 unique genes (22,794 transcripts, Additional files 2 and 3) for which the expression adjusted by the *L*/*G* ratio was significantly different from basal level. (List1) was submitted to gene ontology and enrichment analysis. These analyses showed 106 classes significant at FDR < 0.05. Due to the important number of DEG, generic classes were removed (such as morphogenesis, transcription, locomotion and others). Only genes that were well-known and well described in the literature were chosen to define a final selected list of 284 genes. These genes were grouped into 6 functional classes that were all found enriched for genes (List1) (Immunity and Inflammation, Chemotaxis, Apoptosis, Calcium ion transport, Metabolism, Hormonal Response).

The “immunity and inflammation” class (175 genes) is related to the inflammatory cascade after activation of leukocytes by LPS via TLR4 receptor (a receptor for bacterial lipopolysaccharide). TLR4 is a critical driver of immune responses to bacterial infections. Signals from TLR4 promote NF- *κ*B and AP-1 activation, leading to inflammatory gene expression [28] (DEG for TLR4, TNF, JUNB, and NF-B pathway).

The “chemotaxis” class is composed of 59 genes. Among them *ABHD2*, *ACADS*, *AIF1*, *ANXA7*, *ARPC1A*, *ARPC2*, *CD97*, *CHL1*, *CLIC1*, *CNTFR*, *COQ3*, *DGKD*, *DNASE2*, *GP1BA*, *GPI*, *HCLS1*, *HPS6*, *IL1RN*, *IL8RA*, *KAT5*, *LOC100523056*, *LSP1*, *MAN2B1*, *PARK7*, *PTPN6*, *SMAD7*, *SPG21*, *TMEM173*, *TMSB10*, *TMSB4X*, *TRDMT1*, and *TSPO* genes are related to immune cell trafficking. This observation is in agreement with the observed blood cell redistribution.

The “apoptosis” class (33 genes) includes *C5AR1*, *CCL24*, *CCR1*, *CCR3*, *CXCL13*, *IRG1*, *ALDOC*, *C3AR1*, *CADM1*, *CAPN3*, *HEXA*, *ID3*, *MAEA*, *PLAU*, *PRDX5*, *PROC*, and *CXCR2* genes related to apoptosis and inflammatory response, and *TNFSF13B* and *NFKBIA* involved in cell-activating factor signalling pathway.

Twelve genes (*CD9*, *ANXA5*, *COMT*, *DDIT3*, *ADAM10*, *BAD*, *SOD2*, *ADRB2*, *CLN8*, *LTA*, *TGFBR1* and *PTEN*) form a “calcium ion transport” class.

The “metabolism” class includes four genes (*EDN1*, *COFILIN*, *PLA2G4A*, and *CORO1A*), and the “hormonal responses” class includes one gene (*HMOX1*).

**Fig. 3:**
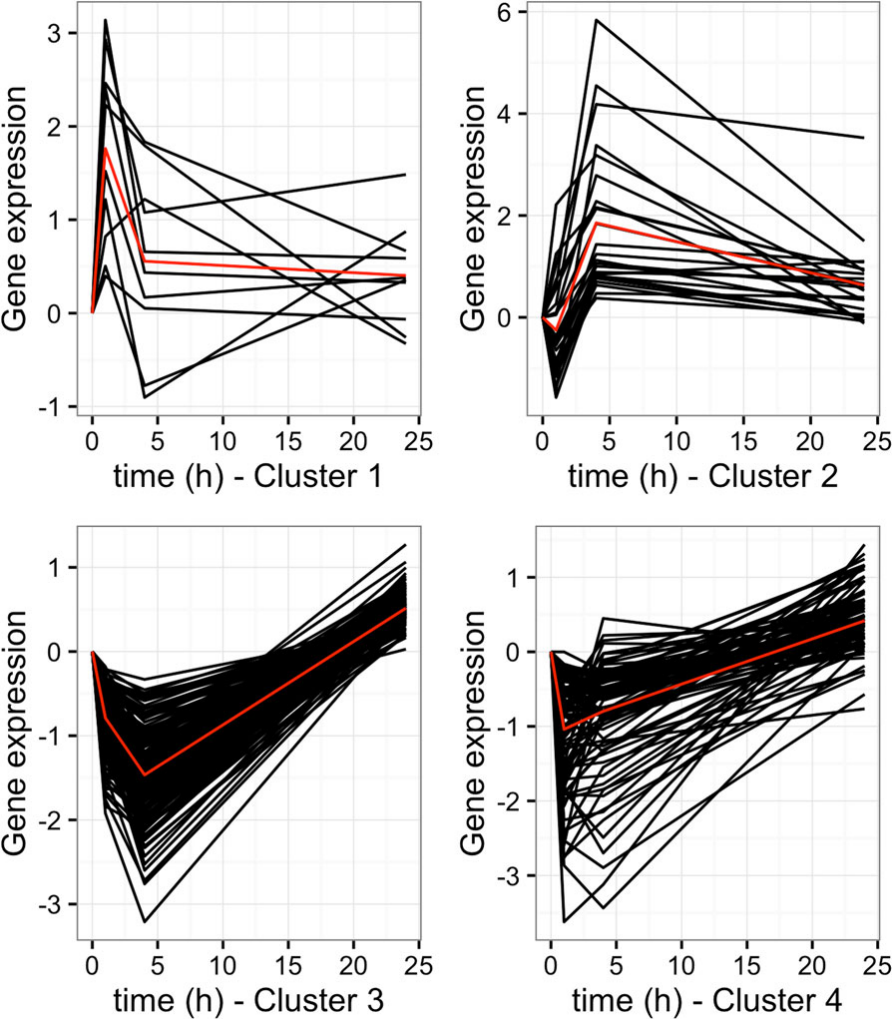
Black: Average evolution the genes in each of the clusters identified by the HAC on the 284 DEG identified in list (**List1**). Evolution of each gene is translated so that it is equal to 0 at *t*=0; Red: Average evolution over all genes in the cluster (cluster 1: 8 genes, cluster 2: 12 genes, cluster 3: 159 genes, cluster 4: 77 genes). 28 genes were unclassified

The 284 remaining genes from the first list (List1) were clustered into 4 clusters using HAC (Fig. 3, Additional file 3).

The **second list of genes (List2)**, consists of 154 unique genes (209 transcripts, Additional file 4) for which the contribution of the *L*/*G* ratio to the expression varied over time steps. Among these genes, 132 genes were further assembled into six functional networks that notably revealed hematological system development and function, tissue morphology, cancer, organismal injury and abnormalities, reproductive system disease, cellular growth and proliferation. The average evolution of all these genes shows the same expression profile. This group of genes is characterized by genes decreasing with a peak of expression at *t*=+1 and stable between *t*=+4 and *t*=+24. According to this analysis, it is unlikely that genes for which the contribution of the *L*/*G* ratio to the expression varied over time steps are directly involved in the immune response to LPS injection.

The **third list of genes (List3)** consists of 116 unique genes (185 transcripts, Additional file 5) for which the expression was found to be correlated to the level of cortisol at *t*=+4. This time point was chosen as the peak of plasma cortisol concentration after LPS (Fig. 1). The most significant functions are: cellular function and maintenance – function of blood cells (30 genes); cellular movement, immune cell trafficking – leucocyte migration (35 genes); lymphoid tissue structure and development, tissue morphology – quantity of lymphatic system cells (34 genes); cellular function and maintenance – function of leucocytes (27 genes); hematological system development and function, tissue morphology – quantity of leucocytes (36 genes); cellular movement, hematological system development and function, immune cell trafficking – cell movement of leucocytes (32 genes); immunological disease – systemic autoimmune syndrome (39,374 genes) (Additional file 6).

### Validation of differential expression by quantitative real-time PCR

Twenty-two DEG were selected for further examination by quantitative real-time PCR using the Fluidigm technique (Table 2). These genes were selected from the three studied lists ((List1)), (List 2), and (List 3)). Pearson correlations between the differences in expression measured by quantitative real-time PCR and microarray were greater than 0.70 for 7 genes (*CHI3L1*, *MYLIP*, *LCK*, *SOD2*, *VAT1*, *COMT*, and *FAS*). Lower correlations (between 0.5 and 0.6) were obtained for *GALK2*, *VNN2*, *JAK2*, *KAT5*, *ADAM10*, *RARA*, and *ANXA7*.

**Table 2:** Correlations between quantitative real-time PCR with microarray expression for selected genes (*n*=22)

Gene name	Gene description	List that provided the gene	Pearson correlation	*p*-value (Fluidigm)	Gene expression profile
*CHI3L1*	chitinase 3 like 1	(List 1)	0.72	1.68e-07	down at *t*=+1
					up at *t*=+24
*CHIT1*	chitinase 1	(List 1)	0.68	0.00214	down at *t*=+1
					up at *t*=+24
*CLEC2D*	C-type lectin domain family 2 member D	(List 2), (List 3)	0.60	0.0019	down at *t*=+1
					up at *t*=+24
*GALK2*	galactokinase 2	(List 1)	0.50	9.52e-11	down at *t*=+1
					up at *t*=+24
*HSD17B11*	hydroxysteroid (17-beta)	(List 1)	0.67	3.02e-11	down at *t*=+1
	dehydrogenase 11				up at *t*=+24
*KAT5*	lysine acetyltransferase 5	(List 1)	0.53	5.37e-09	down at *t*=+1
					up at *t*=+24
*LCK*	LCK proto-oncogene, Src family	(List 1)	0.74	3.77e-10	down at *t*=+1
	tyrosine kinase				up at *t*=+24
*MSN*	moesin	(List 1)	0.61	5.68e-05	down at *t*=+1
					up at *t*=+24
*MYLIP*	myosin regulatory light chain	(List 1)	0.72	2.26e-10	down at *t*=+1
	interacting protein				up at *t*=+24
*RAB31*	RAB31, member RAS oncogene family	(List 1)	0.65	0.00152	down at *t*=+1
					up at *t*=+24
*RARA*	retinoic acid receptor alpha	(List 1)	0.58	6.59e-05	down at *t*=+1
					up at *t*=+24
*SSH1*	slingshot protein phosphatase 1	(List 1), (List 2)	0.67	1.28e-11	down at *t*=+1
		(List 3)			up at *t*=+24
*VAT1*	vesicle amine transport 1	(List 1)	0.75	1.26e-12	down at *t*=+1
					up at *t*=+24
*VNN2*	vanin 2	(List 1)	0.52	0.74	down at *t*=+1
					up at *t*=+24
*CERS4*	ceramide synthase 4	(List 2), (List 3)	0.60	1.16e-06	down at *t*=+1
					up at *t*=+4
*FAS*	Fas cell surface death receptor	(List 1), (List 2)	0.83	<2e-16	down at *t*=+1
		(List 3)			up at *t*=+4
*JAK2*	Janus kinase 2	(List 2), (List 3)	0.52	2.54e-10	down at *t*=+1
					up at *t*=+4
*ADAM10*	ADAM metallopeptidase domain 10	(List 1)	0.56	8.15e-05	down at *t*=+4
					up at *t*=+24
*ANXA7*	annexin A7	(List 1)	0.59	0.000417	down at *t*=+4
					up at *t*=+24
*COMT*	catechol-O-methyltransferase	(List 1)	0.75	1.64e-14	down at *t*=+4
					up at *t*=+24
*STMN1*	stathmin 1	(List 1)	0.69	3.24e-09	down at *t*=+4
					up at *t*=+24
*SOD2*	superoxide dismutase 2,	(List 1)	0.74	<2e-16	up at *t*=+4
	mitochondrial				down at *t*=+24

*p*-value (Fluidigm) gives the time effect of quantitative real-time PCR data for every gene

## Discussion

### Plasma cortisol, metabolites, and blood cell counts

In pigs like in other species, LPS is responsible for the fever and inflammatory reaction induced by gram-negative bacterial infection, as shown by the increase in the circulating levels of pro-inflammatory cytokines and acute phase proteins, as well as the changes in white blood cell counts [27, 29–31], which explained the characteristic changes of white blood cell count to LPS observed in our study.

LPS also induces profound endocrine and metabolic changes and our results are consistent with previously published data in pigs [27, 29, 31]. A large increase in circulating levels of cortisol (and catecholamines, not measured here) has been described and these hormonal changes can be involved in the release of the mediators of inflammation [27, 31]. It was shown previously in mice that this hypoglycaemia cannot be explained by changes in insulin concentrations that are also reduced by LPS [32], but it could result from the increased glycolysis in muscles and immune cells, as well as from a reduced hepatic glucose production [33]. The increase of circulating concentration of free acids can result from the lipolytic action of catecholamines and cortisol that are massively released by LPS [11, 34] and from LPS-induced changes in hepatic and fat tissue lipid metabolism [35, 36]. A sharp increase in bilirubin concentrations was also measured at *t*=+4 (17.72 vs 2.14 *μ*mol/l), reflecting the hepatic toxicity of LPS [37, 38].

### Clustering of differentially expressed genes (List1)

The 284 remaining genes from the first list (List1) were clustered into 4 clusters using HAC. This clustering exhibited patterns related to different kinetics of their response.

**The first cluster** includes 8 genes up-regulated at *t*=+1 and related to immune cell tracking (*ALOX12*, *JUNB*, *TNFAIP3*, *CCL20*, *CXCL5*, *NFKBIA*, *LTA*, *EDN1*). Inflammation is a powerful protective mechanism which is coordinated and controlled by cytokines and chemokines and, as expected, we detected an increase in the expression level of members of the CXCL family. *JUNB* gene (a member of Jun family) also participates in the immune response; it is activated by the TLR signalling pathway [39] and can induce expression of interleukins [40–43]. Hormone activation of the glucocorticoid receptor in leukocytes results in a profound suppression of pro-inflammatory gene networks such as the NF- *κ*B mediated transcription of pro-inflammatory cytokine genes and *CXCL2* together with *LTA* were described by [44] as glucocorticoid-regulated genes. These findings show that wide variation in glucocorticoid sensitivity exists between individuals which may influence susceptibility to inflammatory diseases [11].

**The second cluster** includes 12 genes up-regulated at *t*=+4 and related to connective tissue disorders and inflammatory diseases (*GYG1*, *PDXK*, *RETN*, *C3*, *IL27*, *TLR4*, *IL1RN*, *ICAM1*, *CXCL13*, *C3AR1*, *FAS*, *SOD2*, and *TLR4*). Toll-like receptor 4 (*TLR4*) is essential for initiating the innate response to lipopolysaccharide from Gram-negative bacteria by acting as a signal-transducing receptor. As the pig industry faces a unique array of related pathogens, it is anticipated that the genotype of swine *TLR4* could be of crucial importance in future strategies aimed at improving genetic resistance to infectious diseases [45].

**The third cluster** includes the genes down-regulated at *t*=+4. This cluster groups 159 DEG related to the inflammatory response. It is associated with functions linked to immunological disease, cancer, cell death and survival, immune cell tracking, and belongs to a series of twelve canonical pathways, including leukocyte extravasation signalling, NF- *κ*B activation, and glucocorticoid receptor signalling.

**The fourth cluster** includes the genes down-regulated at *t*=+1. These genes are related to apoptosis, NF- *κ*B, and death receptor signalling canonical pathways.

### Comparison of all lists of genes

**Fig. 4:**
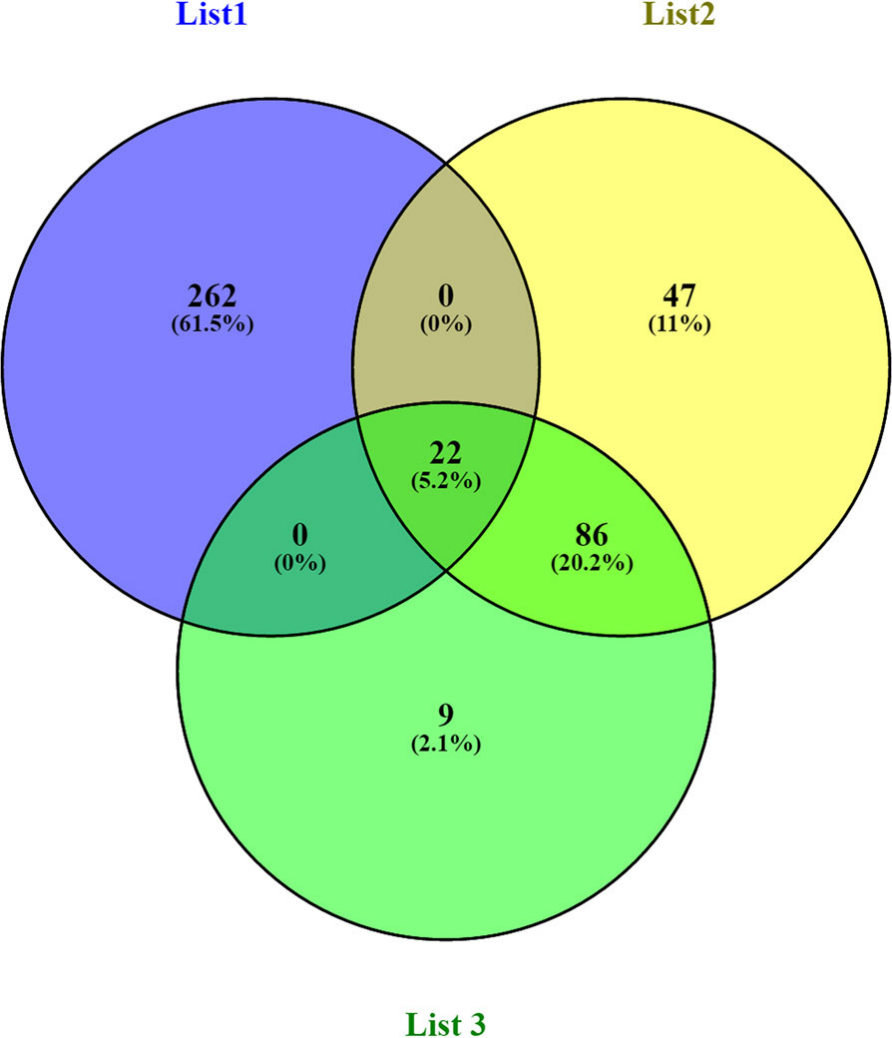
Venn diagram showing DEG common to all lists of genes ((List1), (List12 and (List3); 22 DEG)

**Fig. 5:**
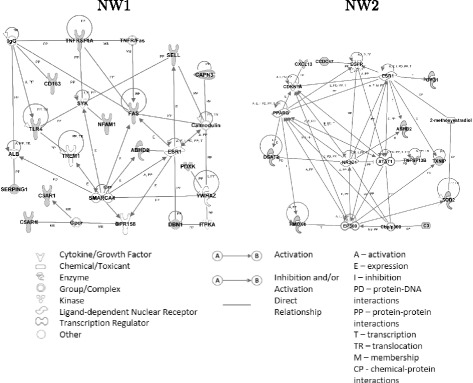
Functional networks formed by the 22 DEG common to (List1), (List2) and (List3). NW1: related to infectious diseases, cellular movement, hematological system development and function, cell-to-cell signalling and interaction. NW2: related to cell death and survival, cellular development, cellular growth and proliferation

Figure 4 shows the overlap of the three lists of genes ((List1), (List2), and (List3)). Twenty two genes are common between the three analyses: *ABHD2*, *C3*, *C3AR1*, *C5AR1*, *CAPN3*, *CCDC47*, *CD163*, *CXCL13*, *DBN1*, *DGAT2*, *FAS*, *GYG1*, *HMOX1*, *NFAM1*, *PDXK*, *SELL*, *SERPING1*, *SOD2*, *TLR4*, *TNFRSF1A*, *TNFSF13B* and *TXNIP*. Identification of genes common to all three analyses, which are known in literature to have an important role in chemotaxis, apoptosis, calcium ion transport and metabolism, confirms their roles in immunity and inflammation in pigs. These genes could further serve in a panel of tissue prognosis indicators of porcine immune response. IPA analysis showed that these genes form two functional networks, NW1 and NW2 (Table 3). The first functional network (NW1, Fig. 5) is related to infectious diseases, cellular movement, hematological system development and function, cell-to-cell signalling and interaction. These genes form a node connected to *ESR1*. This gene encodes the estrogen receptor 1, a ligand-activated transcription factor. Estrogen receptors are also involved in pathological processes including breast cancer, endometrial cancer, and osteoporosis [46]. Among the genes that form these networks, two are particularly interesting, *TLR4* and *CD163*. Toll-like receptor 4 (*TLR4*) signalling pathway is the essential member in TLRs family, which plays an important role in a variety of inflammatory reaction such as in diarrhoea and hydropsy of weaned piglets infected by pathogens [47]. The *TLR4* gene was described as one of the important immunological factors influencing for example the development of mycoplasma pneumonia of swine [48]. TLR4 dysregulation promoted aberrant cytokine production in bacterial sepsis [49].

**Table 3:** Gene networks (NW) with commons DEG between the three lists of genes (in bold)

NW	Genes in network	Genes in present study	Top diseases and functions
1	***ABHD2*** ^∗^, *ALB*, ***C3AR1***, ***C5AR1***, *Calmodulin*, ***CAPN3***, ***CD163***, ***DBN1***, ESR1*, ***FAS***, *Gpcr*, *GPR158*, *IgG*, *ITPKA*, ***NFAM1***, ***PDXK***, ***SELL***, ***SERPING1***, *SMARCA4*, *SYK*, ***TLR4***, *TNFR/Fas*, ***TNFRSF1A***, *TREM1*, *YWHAZ*	13	Infectious Diseases, Cellular Movement, Hematological System Development and Function, Cell-To-Cell Signaling and Interaction
2	*2-methoxyestradiol*, ***ABHD2*** ^∗^, ***C3***, *Cbp/p300*, ***CCDC47***, *CDKN1A*, ***CXCL13***, ***DGAT2***, *EGFR*, *EP300*, ESR1*, ***GYG1***, ***HMOX1***, *NR3C1*, *PPARG*, ***SOD2***, *STAT1*, ***TNFSF13B***, ***TXNIP***	10	Cell Death and Survival, Cellular Development, Cellular Growth and Proliferation

*ABHD2* ^∗^ and *ESR1* ^∗^ are common to both networks

The expression of porcine *CD163* (a scavenger receptor belonging to a cysteine-rich superfamily) on monocytes/macrophages correlates with permissiveness to African swine fever infection [50]. *CD163* is considered as the most important receptor for porcine reproductive and respiratory syndrome attachment and internalization [51]. Cell entry of simian hemorrhagic fever virus is also dependent on *CD163* [52].

The second network (NW2) is related to cell death and survival, cellular development, cellular growth and proliferation. The *NR3C1* (nuclear receptor subfamily 3, group C, member 1) gene encodes the glucocorticoid receptor, which can function both as a transcription factor that binds to glucocorticoid response elements in the promoters of glucocorticoid responsive genes, and as a regulator of other transcription factors. Signal transducer and activated transcription 1 (*STAT1*) has been identified as a point of convergence for the cross talk between the pro-inflammatory cytokine interferon *γ* (IFN*γ*) and the Toll-like receptor-4 (*TLR4*) ligand LPS in immune cells [53]. LPS activates *STAT1* via the NF-*κ*B pathway [54].

Several transcriptomic studies of immune and inflammatory responses have been published in pigs, however little is known about longitudinal changes. The peripheral blood transcriptome reflects variations in immunity traits and a few potential gene biomarkers were found for immunocompetence (*RALGDS* gene was shown for prediction of IL2; *ALOX12* for phagocytosis; *GNLY*, *KLRG1* and *CX3CR1* for CD4-/CD8+ cell count) [55]. Zhou et al. [56] investigated the transcriptional responses of pig peripheral blood mononuclear cells following an experimental challenge with the intracellular protozoan *Toxoplasma gondii*. Zhao et al. [57] studied the response to the foot-and-mouth disease infection. Peripheral blood mononuclear cells transcriptome profiles were studied by Islam et al. [58] to identify potential candidate genes and functional networks controlling the innate and adaptive immune responses to the porcine reproductive and respiratory syndrome vaccine. The innate immune transcriptional network was found to be regulated by *LCK*, *STAT3*, *ATP5B*, *UBB* and *RSP17* genes. The adaptive immune transcriptional response to the porcine reproductive and respiratory syndrome vaccine in peripheral blood mononuclear cells is related to *TGFb1*, *IL7R*, *RAD21*, *SP1* and *GZMB*. Altogether these results show the value of gene expression studies to explore inflammatory and immune responses and the factors of their regulation.

## Conclusion

We have presented here an integrative biological approach combining different statistical models and biological measures and taking into consideration the longitudinal aspect of the data. This analysis of biological data required the development of a methodology adapted to both the multi-dimensional and longitudinal data.

LPS stimulation was chosen because it is standard to study general inflammation processes in many species. Immunological stress is the status of animals challenged by bacteria or viruses. It is associated with immunological, neurological, and endocrinological responses [4]. A four time point kinetic was studied. It has been reported that time points earlier than 24 h are more relevant to decipher the onset of the response to stimulus as shown in kinetics studies in cow [59], pig [60], mouse [61] or human [62]. Moreover, kinetic studies have revealed that many genes return to their basal expression level by 24-48 h of stimulation, suggesting that homeostasis is restored at that time [59, 60]. Our results provide many candidate genes to test for kinetic studies and ongoing complementary studies focused on this topic. It is worth mentioning that the different responses to LPS are not influenced by the adrenal gland reactivity as measured by the cortisol response to ACTH.

In conclusion, we have demonstrated that there are specific biomarkers indicative of an LPS-stimulated inflammatory response. Furthermore, these responses persist for prolonged periods of time and at significant expression levels, making them good candidate markers for evaluating the efficacy of anti-inflammatory drugs. The majority of the genes identified have known roles in the inflammatory process. Subsequently, these biomarkers may serve collectively as an indication of inflammation in swine. The knowledge gained from this series of experiments may help in the development of a model for further studies.

## Additional files

Additional file 1 Sequences of oligonucleotide primers tested by quantitative RT-PCR analysis. Gene name: name of the gene; Gene description: informations on the gene’s molecular function; Forward Primer; Reverse Primer. (XLSX 11 kb)

Additional file 2 List of 9,530 unique genes differentially expressed in list (List1). (XLSX 2360 kb)

Additional file 3 List of 284 unique genes differentially expressed in list (List1) included in non-generic biological functions (*n*=30). Generic biological functions include functions such as morphogenesis, transcription, locomotion. Gene name: name of the gene; Gene description: informations on the gene’s molecular function; Cluster: cluster in which the gene is classified by HAC; Time point: time measurement where the gene is DE; Expression: whether the DEG is up or down-regulated. (XLSX 21.2 kb)

Additional file 4 List of 154 unique genes differentially expressed in list (List2). (XLSX 35.1 kb)

Additional file 5 List of 116 unique genes differentially expressed in list (List3). (XLSX 22.9 kb)

Additional file 6 Biological functions enriched by differentially expressed genes in list (List3) (*n*=30). Categories: Category of the enriched function; Diseases or Function Annotation: name of the enriched function; FDR: FDR of the enrichment test; Genes: list of genes enriching the biological function; Genes: number of genes enriching the biological function. (XLSX 10 kb)
